# Coexistence of Insect Species Competing for a Pulsed Resource: Toward a Unified Theory of Biodiversity in Fluctuating Environments

**DOI:** 10.1371/journal.pone.0018039

**Published:** 2011-03-21

**Authors:** Samuel Venner, Pierre-François Pélisson, Marie-Claude Bel-Venner, François Débias, Etienne Rajon, Frédéric Menu

**Affiliations:** 1 Université de Lyon, Lyon; Université Lyon 1; CNRS, UMR5558, Laboratoire de Biométrie et Biologie Evolutive, Villeurbanne, France; 2 Department of Ecology and Evolutionary Biology, University of Arizona, Tucson, Arizona, United States of America; University of Utah, United States of America

## Abstract

**Background:**

One major challenge in understanding how biodiversity is organized is finding out whether communities of competing species are shaped exclusively by species-level differences in ecological traits (niche theory), exclusively by random processes (neutral theory of biodiversity), or by both processes simultaneously. Communities of species competing for a pulsed resource are a suitable system for testing these theories: due to marked fluctuations in resource availability, the theories yield very different predictions about the timing of resource use and the synchronization of the population dynamics between the competing species. Accordingly, we explored mechanisms that might promote the local coexistence of phytophagous insects (four sister species of the genus *Curculio*) competing for oak acorns, a pulsed resource.

**Methodology/Principal Findings:**

We analyzed the time partitioning of the exploitation of oak acorns by the four weevil species in two independent communities, and we assessed the level of synchronization in their population dynamics. In accordance with the niche theory, overall these species exhibited marked time partitioning of resource use, both within a given year and between different years owing to different dormancy strategies between species, as well as distinct demographic patterns. Two of the four weevil species, however, consistently exploited the resource during the same period of the year, exhibited a similar dormancy pattern, and did not show any significant difference in their population dynamics.

**Conclusions/Significance:**

The marked time partitioning of the resource use appears as a keystone of the coexistence of these competing insect species, except for two of them which are demographically nearly equivalent. Communities of consumers of pulsed resources thus seem to offer a promising avenue for developing a unifying theory of biodiversity in fluctuating environments which might predict the co-occurrence, within the same community, of species that are ecologically either very similar, or very different.

## Introduction

Ecologists have been intrigued for decades by how competing species coexist in ecological communities [1]–[4], and they have proposed two radically opposed theories to explain species diversity. The niche theory suggests that the dynamics and structure of ecological communities are mainly attributable to differences in ecological traits between species (niche partitioning). These differences, which have the effect of lowering interspecific relative to intraspecific competition, may favor the coexistence of several species by giving any one of them a greater propensity to increase when it is rare than when it is common [2], [5]–[6]. This density-dependent property might promote the stable coexistence of competing species whenever it overcomes differences in the species' competitive ability or demographic performance [2]. In contrast, the neutral theory of biodiversity hypothesizes that trait differences between species do not structure ecological communities. Accordingly, neutral models assume that competing species are all ecologically similar (*i.e.* their demographic parameters are equivalent on average), and predict that the structure of communities will be primarily shaped by demographic stochasticity, which is itself derived from random differences between individuals within the community with regard to their survival, reproduction and migration (unstable coexistence or co-occurrence) [3], [7]. Recently, several reviews and theoretical developments have promoted a unifying theory of biodiversity [8]–[15]. Some of them suggest a continuum of hypotheses, between those considering either a strong niche differentiation or on the contrary the ecological similarity between competitive species, to explain species diversity [13], [15]. One major concern in ecology is whether species diversity mainly results from strong niche differences that stabilize the coexistence of highly unequal competitors, or from low niche differences that are sufficient to stabilize the interactions between nearly-equivalent competitors, or even from a mixed situation between these two extremes.

Finding empirical evidence for these theories is a complex task, but focusing on communities displaying marked temporal variations in the availability of resources (*e.g*., variations over time of water in desert ecosystems [16]) might provide a promising way to test them, as in fluctuating environments each theory leads to very different predictions that can be tested over short periods [17]. According to the niche theory, species differ in their ecological niche (*i.e.* in their response to the varying environment), and this can be expected to lead to asynchronous dynamics, which should be particularly easy to detect when environmental variation is high [2], [17], [18]. In contrast, the neutral theory of biodiversity assumes that all competing species are demographically equivalent. No niche partitioning is particularly expected between them, and they are expected to be equally affected by environmental fluctuations over time. Accordingly, any growth or decline of their population should be synchronized over a short timescale [17].

These divergent predictions make it possible to investigate the mechanisms governing the co-occurrence of specialist species exploiting the same recurrent pulsed resource [18]–[20]. A resource is pulsed when it sporadically becomes highly abundant, but remains scarce during variable intervals of time [21]. Pulsed resources are relatively common [21], and seem to be suitable for use to explore the mechanisms of competitive coexistence. Nevertheless, empirical studies based on such systems are scarce, and most have so far been restricted to desert plants with population dynamics driven by the availability of water [16], [22]–[25]. These studies provide elements consistent with a stabilizing coexistence mechanism via marked niche partitioning: some plant species, known to have drastically divergent traits, respond in different ways to water pulses and display asynchronous population dynamics [16], [18]. Since systems relying on pulsed resources are especially relevant for exploring the theories of biodiversity, it is becoming critical to expand our knowledge to include communities of consumers of such resources. Masting is a reproductive strategy exhibited by some perennial plants that consists of the intermittent production of large seed crops synchronized at the level of the population [26]. Seeds produced by these plants constitute a recurrent, pulsed resource for their specialist parasites or predators. Many insect species are known to exploit such mast-seeding plants, mostly during their larval development, and to co-occur at the individual tree level [27]–[28]. However, the processes underlying their co-occurrence have yet to be studied.

The main objective of our study was to test mechanisms that might promote the local coexistence of phytophagous insect species specialized on oak trees (*Quercus sp*.) that are known to exhibit mast-seeding strategies [29]–[30]. In the geographical area studied, four sister species of weevil (*Curculio glandium* (Marsham), *C. elephas* (Gyllenhal), *C. pellitus* (Boheman) and *C. venosus* (Gravenhorst)) co-occur on the same trees, where they infest oak acorns for their larval development [31]–[32]. Adult weevils breed only during the summer following their emergence. Eggs are laid within oak acorns; larvae then develop fully within the host fruit before leaving it and burrowing into the soil. Adults emerge from the soil between one and four years later, depending on the variable duration of the diapause [32]–[34]. Depending on whether these weevil communities are primarily shaped by strong stabilizing processes or, conversely, if the weevil species are demographically nearly equivalent, very divergent predictions will be made.

Several stabilizing processes may have the effect of promoting the coexistence of the four insect species, but they are likely to be tightly linked to the marked fluctuations in resource availability that have a major impact on the dynamics of insect populations. We focused on the storage effect, which is considered to be a major mechanism underlying stable coexistence in variable environments [2], [18]. There are 3 pre-requisites for a storage effect: (i) the species must differ in their response to the varying environment (temporal niche partitioning); (ii) they must have high levels of persistence during the times when they are not favored (buffered population growth); and (iii) the strength of competition must vary with environmental conditions (thus precluding competitive exclusion by allowing species to recover from low density levels while impeding the growth capacity of more common species). In the insect communities we studied, the “buffered population growth” (*i.e.*, the second pre-requisite of the storage effect) may come about during the dormancy phase experienced by weevil larvae, and during which very low mortality rates are observed [35], [36]. During this stage, all four weevil species experience similar environment conditions, since all mature larvae build an individual pen underground where they overwinter at a lowered metabolic rate and without feeding [35]. Consequently, we instead focus on any between-species differences related to the acorn exploitation. From a theoretical investigation of the storage effect operating on communities competing for a pulsed resource [18], it is likely that time partitioning of the resource use (the first pre-requisite), which might lead environmental conditions and competition to covary (the third pre-requisite), will operate at two different timescales (see the detailed illustration in Chesson *et al*. [18]):

(i) the competing species might exhibit different dormancy durations, leading to a between-year partitioning of the resource use. The decision for one insect to pursue its dormancy or to embark on an active life in a given year has to be made long before it uses the resource, and does not seem to rely on any clue predicting its future availability and/or quality. Moreover, years of high pulses of the resource are to some extent unpredictable. Such randomly alternating years of high and low resource availability, combined with specific dormancy strategies, might favor each of the competing species in turn, and thus stabilize their coexistence [37]. Hence, a rare species emerging during a year of high resource abundance would suffer little from competition either within the species (because of its low population density) or between species (since the other species do not emerge simultaneously), and thus should experience a relatively high growth rate. Conversely, an abundant species emerging in a favorable year might suffer from intraspecific competition, and therefore display limited growth, which would in turn restrict its negative impact on other species.

(ii) the species might experience within-year time partitioning, with some of them being able to exploit green acorns while others, exploiting acorns later in the breeding season, develop on mature fruits. Early-laying species should be at an advantage, as compared to late-laying species, because they have prior access to the oak acorns. Such a strategy should work best when competition is high (*e.g.* when there is a high density of ready-to-lay females but low acorn availability). In turn, species present later in the season, and that avoid already infested oak acorns (unpublished data), would be favored if they exploit fruits more efficiently than earlier-emerging species. This higher efficiency could be due to their ability to lay eggs in already mature and uninfested acorns, which promotes full larval development since eggs of the earlier-laying species may never develop if laid in aborting fruits. Species laying late in the season would therefore have an advantage over early-laying species when the competition for acorn exploitation is low (*e.g*., with a large seed crop and a low density of breeding adults). Over several years, each species might be favored in turn owing to variations in the degree of competition for the resource linked to the fluctuating availability of the resource.

Conversely, if insect community structures mainly result from neutral processes, these four species should be demographically nearly equivalent. No particular time partitioning would be expected between them. They would be equally affected by the strong temporal fluctuations of acorn availability; as a result, their population dynamics would be synchronized over time. To explore the mechanisms that might promote species coexistence in these insect communities, we investigated the timing of resource use within and between years for each weevil species. In addition, we explored whether some or all of these species display synchronized dynamics. We studied these characteristics in two distinct natural communities of oak weevils (*Curculio* spp.).

## Materials and Methods

We studied insect communities composed of four weevil sister species of the genus *Curculio* (*C. glandium*, *C. elephas*, *C. pellitus*, *C. venosus*) that infest acorns on the same individual oak trees. We focused on isolated oak trees, located in a fragmented, agricultural landscape, and given that weevils are known to be poor dispersers [36], we assumed that all the weevils found on the same tree belonged to one community. This hypothesis was also supported by the fact that distinct genetic structures were found in one of the weevil species, *C. elephas*, between populations located within 200 m of one another (unpublished data). We surveyed two such communities located 40 km apart near Lyon (France). In each locality we selected one tree between 150 and 200 years of age (tree A (*Quercus robur*): N45° 35′; E5° 01′; tree B (*Q. petraea*): N45° 45′; E5° 16′).

### Oak acorns as a limiting resource

To find out whether the availability of oak acorns can be limiting some years for the weevil species, and whether this might impede their population dynamics, we investigated the relationship between the annual acorn crop of each tree during five consecutive years (2004–2008) and 1) the number of mature larvae emerging from these oak acorns or 2) the competition strength between weevils for this resource. To do this, every day throughout the acorn season, *i.e*. from mid-August to the end of December, we harvested all the mature acorns that had dropped off each tree onto a net placed on the soil over half the area under the tree. These acorns were then placed in wire-netting boxes in an outdoor arena that allowed us to collect and count all the mature weevil larvae that had emerged naturally on a daily basis. This sampling procedure was repeated yearly for each tree during the five-year survey. The degree of competition between the oak weevils was estimated yearly at each tree from the proportion of oak acorns attacked by weevils. We considered a fruit to have been attacked by adult weevils whenever scars were visible on its surface, reflecting one or more egg-laying attempts, and/or exit holes characteristic of weevil larvae were visible at the end of the experiment.

### Time partitioning across years

To test whether species exhibit between-year time partitioning of their resource use, all the emerging mature larvae collected daily during a given year at a given tree were randomly assigned to several covered, water-permeable plastic receptacles that had previously been filled with sifted soil and partially buried under each host tree. The weevils were allowed to burrow freely. We then routinely performed a weekly survey of adult emergence throughout the four years after the larvae had developed and buried themselves. As soon as an adult was detected in one of the receptacles, we started a daily survey that ended when no further adult emerged during at least eight consecutive days in either receptacle. Once the four-year survey had ended, all the receptacles were excavated and carefully examined, but we never found any living weevil still buried after that time. We repeated the experiment from 2004 to 2006 at tree A, and in 2005 at tree B. The larvae from each cohort and each tree were placed in separate receptacles. We failed to survey the larval cohorts that developed on tree B in 2004 and in 2006, either because the experimental device was damaged (cohort 2004), or there were too few larvae to set up the experiment (2006). We tested the partitioning of the resource use by the four weevil species by comparing the distribution of adult emergence over four years.

### Time partitioning within a year

We surveyed the two insect communities by sampling adult weevils on both trees every year from 2004 to 2007. This sampling was limited to the egg-laying period (*i.e.*, the period when acorns were being exploited), that is, from the time when the first egg-laying scars were observed on the surface of the acorns (early June) up to the time when no adult was found on either tree at two consecutive sampling sessions (mid-October). To ensure that the sampling effort was consistent throughout the sessions, we randomly selected 6 and 7 branches on trees A and B, respectively, and these same branches were sampled throughout the breeding season and from one year to the next. We used the beating/sheet method since this is mostly appropriate for collecting, early in the day, insects that play possum when surprised [38]. Within an hour after sunrise each branch was beaten downwards three times, at a distance of 2 to 3 meters from the trunk, and the insects that fell off were collected on a white sheet previously laid out under the branch. This sampling enabled us to identify the sex and the species of each weevil collected and to determine, for each species, the timing of its resource exploitation, and to estimate its annual abundance. The time partitioning throughout the breeding season was computed irrespective of sex for each species.

### Relative abundance of the four weevil species and synchrony of their population dynamics

From the adult sampling described in the previous section, we computed the total number of adults per species per year, which enabled us to compare the relative abundance of the four weevil species surveyed yearly from 2004 to 2007 in each of the two communities, and to test the degree of synchrony of the population dynamics between these species (*i.e.* the correlation between the sizes of their populations).

### Data analysis

#### Oak acorn as a limiting resource

**Figure 1:**
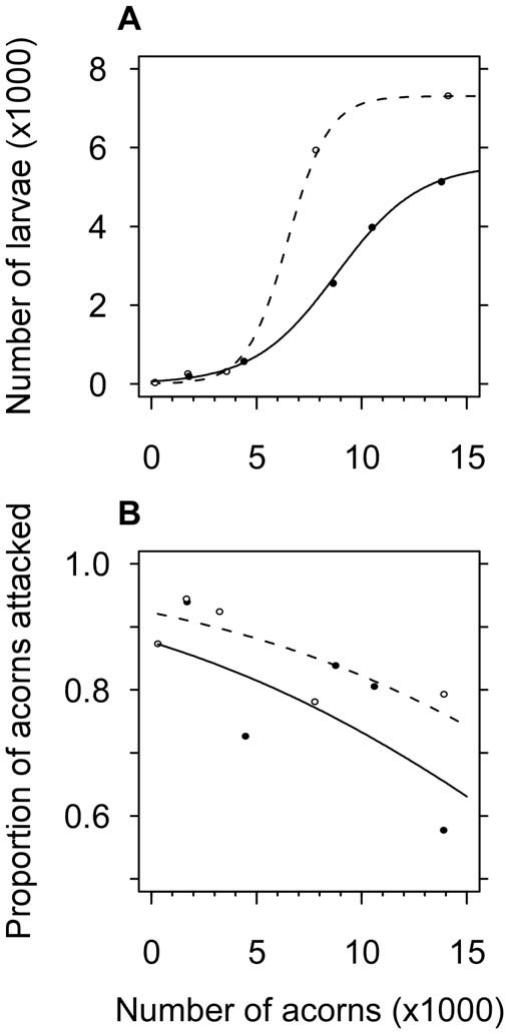
Oak acorn as a limiting resource. The number of mature larvae (A) and the proportion of acorns attacked by weevils (B) in either tree depend on the number of acorns produced by that tree. Samples were obtained each year from 2004 to 2008 on Tree A (dotted line, open circles) and Tree B (plain line, full circles). The logistic curves illustrate the best-fitted models predicting the number of larvae from the acorn crop at each tree, respectively.

we used a linear mixed model including the tree and the year as random effects to test the relationship between the number of acorns produced and the logit-transformed number of mature larvae (see Figure 1 for the logistic relationship between the number of acorns and the number of larvae). To test the relationship between the number of oak acorns produced a given year, and the probability that an oak acorn would be attacked by weevils, we used a logistic mixed model, with the tree as a random effect.

#### Time partitioning

To explore the time partitioning of the resource use between the four weevil species at the two different timescales we used generalized linear mixed models with a Poisson distribution of error. For the between-year time partitioning, the predicted variable is the number of adults of each species that emerged during each of the four years following larval burrowing. In this analysis, we considered the tree and the cohort (*i.e.* the year of larval development) to be random effects. We considered the year of emergence following larval development and the species to be fixed effects. To detect between-year time partitioning we tested whether the interaction between the species and the year of emergence significantly explained the number of adults emerging.

We used the same type of model to detect within-year partitioning. We aimed to predict the number of adult weevils observed on an oak tree during a breeding season. In our model, we considered the tree and the year of adult sampling to be random effects. The species and the week during the breeding season when an adult was found on a tree were both fixed effects. We tested whether the interaction between the species and the week of adult presence significantly explained the number of adults found on a tree. The years 2007 and 2008 were not illustrated because too few weevils of some species were sampled (n≤3) and thus, these data were not representative of the time interval of the actual resource use by these species during the breeding period.

#### Synchrony of the dynamics

To detect asynchronous dynamics between the species, we analyzed the two trees separately, and tested the between-year variations in the relative abundance of the four species with Chi-squared homogeneity tests. Then, to find out whether any of the species were synchronized, we tested the correlation between the population size of two species with an ANCOVA, including the trees as the factor.

In all the analyses, data included both males and females. Because it was the females which were laying in the oak acorns, we repeated the data analysis considering females only. We always found similar results (both qualitative and quantitative) regardless of whether the two sexes were considered together or only the females (data not shown).

## Results

### Oak acorns as a limiting resource

The number of acorns produced was found to vary considerably from one year to the next at both of the trees studied (Figure 1). Oak acorns seem to be a limiting resource for the specialist weevil species studied for two reasons. First, the number of weevil larvae that achieved their development on one tree a given year was strongly and positively related to the number of acorns produced by that tree during that year (Figure 1A; F_1,4_ = 11756, p<0.0001). Second, oak acorns were attacked at a higher rate by weevils when they were produced in smaller numbers, *i.e.* the probability than an oak acorn would be attacked in a given year decreased as the number of acorns produced by that tree increased (Figure 1B; z = 5.17, *p*<0.0001). Taken together, these findings suggest that there was greater competition between weevils for larval development in the years when the number of oak acorns produced by a given tree is low.

### Time partitioning across years

**Figure 2:**
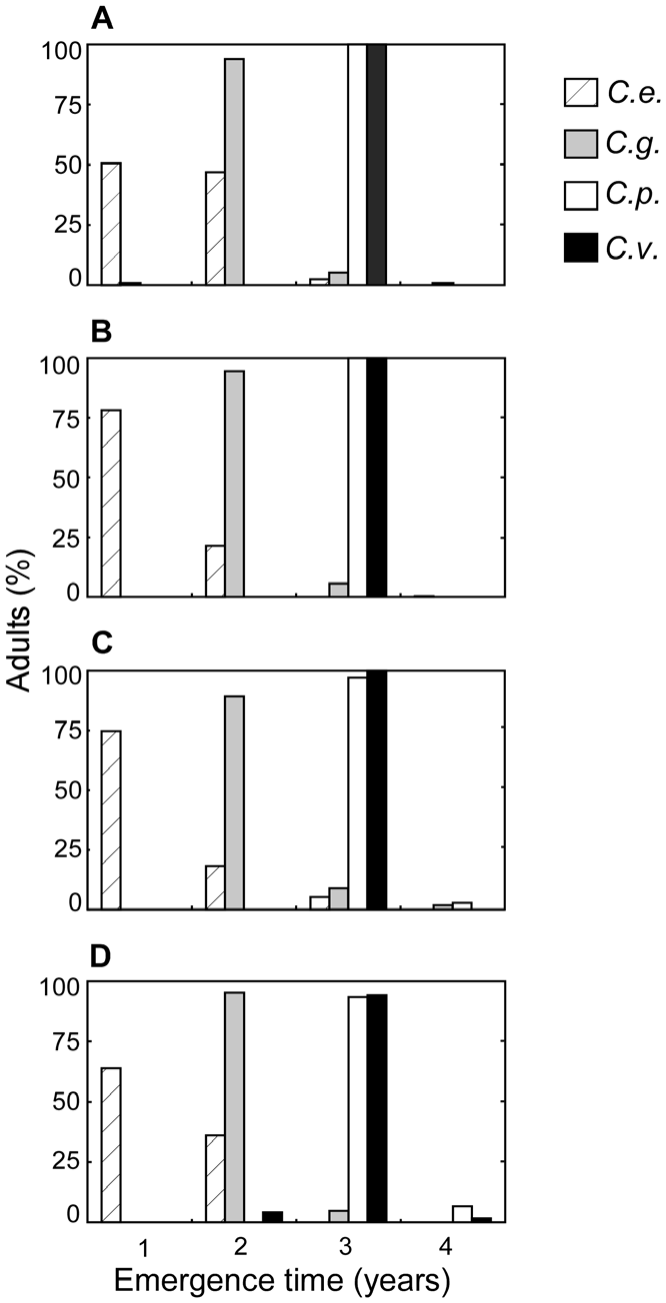
Time partitioning of the resource use between years by the four weevil species. *C. v*.: *Curculio venosus*; *C. p*.: *C. pellitus*; *C. g*.: *C. glandium*; *C. e*.: *C. elephas*. Following larval development at one tree a given year, we made a four-year survey of adult emergence and computed for each species their distribution across years. We found a significant, consistent time partitioning across years by the four species, following larval emergence at Tree A (**A**) in 2004, (**B**) in 2005, (**C**) in 2006 and (**D**) at Tree B in 2005. Only *C.v.* and *C.p.* never showed any difference in their distribution at any tree and for any larval cohort.

We found that the four weevil species overall exhibited clear time partitioning of resource use. At both trees we found consistent partitioning of the resource use between years by the four *Curculio* species (Figure 2, test of the interaction between the species and the year of adult emergence following larval development: 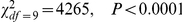). *C. elephas* mainly emerged during the first two years following larval development; *C. glandium* massively emerged almost exclusively during the second year, while both *C venosus* and *C. pellitus* almost exclusively emerged during the third year. We did not find any significant difference in the distribution of adult emergence across the years between *C. venosus* and *C. pellitus*, whereas highly significant differences were found in all other pairwise comparisons (Figure 2, Table 1).

**Table 1:** Analysis of the time partitioning between years, and within the year, of the four *Curculio* species, and correlation between the size of their populations across years.

	*C. glandium*			*C. pellitus*			*C. venosus*		
	Between-year time partitioning								
	df	Khi-square	*P*	df	Khi-square	*P*	df	Khi-square	*P*
*C. elephas*	3	2203	<10e-15	3	378	<10e-15	3	1659	<10e-15
*C. glandium*				3	297	<10e-15	3	1486	<10e-15
*C. pellitus*							3	5.28	0.15
	Within-year time partitioning								
	df	Khi-square	*P*	df	Khi-square	*P*	df	Khi-square	*P*
*C. elephas*	21	240	<10e-15	21	296	<10e-15	21	572	<10e-15
*C. glandium*				21	121	<10e-15	21	372	<10e-15
*C. pellitus*							21	23.4	0.32
	Correlation between the size of the populations across years								
	df_1_, df_2_	F	*P*	df_1_, df_2_	F	*P*	df_1_, df_2_	F	*P*
*C. elephas*	3, 4	2.9	0.17	3, 4	0.21	0.88	3, 4	0.43	0.74
*C. glandium*				3, 4	0.14	0.43	3, 4	0.87	0.28
*C. pellitus*							3, 4	37.5	0.002

### Time partitioning within the year

**Figure 3:**
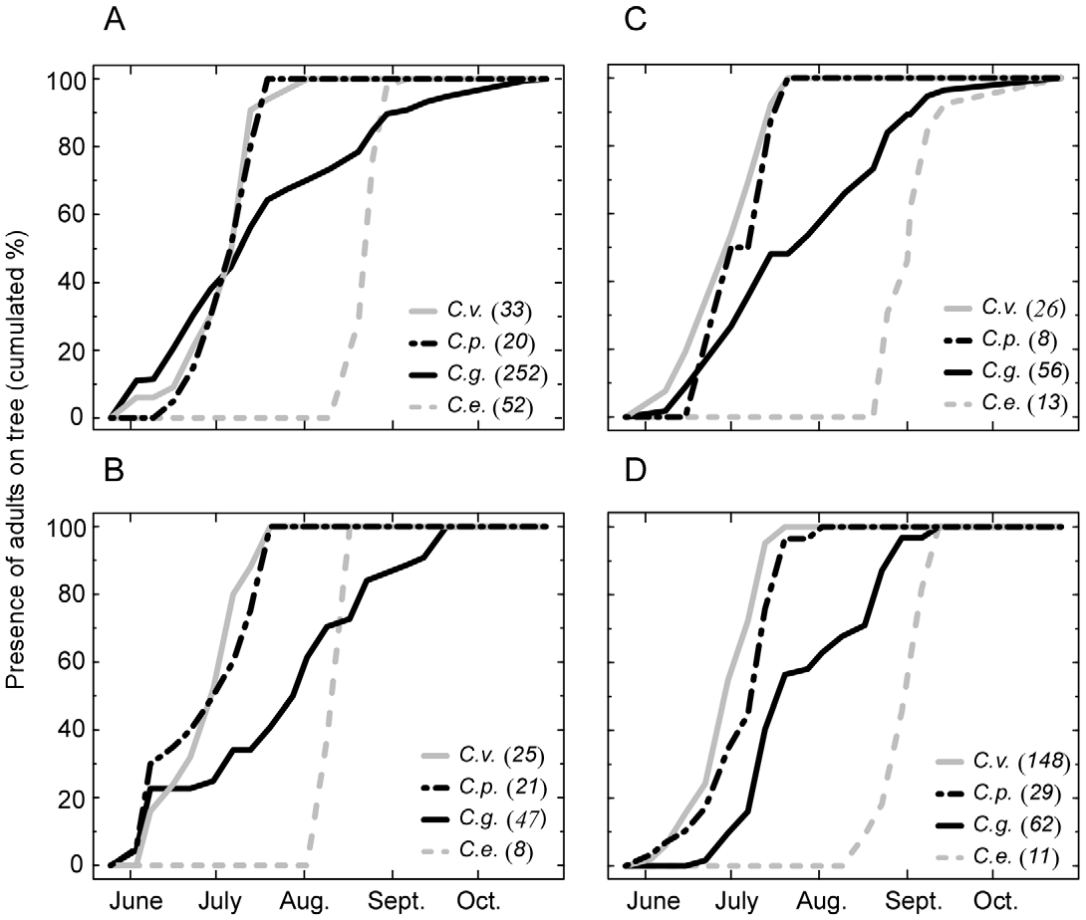
Time partitioning of the resource use within the year by the four weevil species. The cumulative abundance over the breading season of adults of each species is shown for weevils sampled on Tree A in 2004 (A) and 2005 (B), on Tree B in 2004 (C) and 2005 (E). *C. v*.: *Curculio venosus*; *C. p*.: *C. pellitus*; *C. g*.: *C. glandium*; *C. e*.: *C. elephas*. The four weevil species overall exhibited a strong time partitioning of their resource exploitation. Two of the four weevil species, *C. venosus* and *C. pellitus*, however, used the resource during the same period of the year.

We found marked within-year partitioning (Figure 3, test of the interaction between the species and the week of presence of adults on the trees during the breeding season: 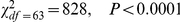). In these four species, we can identify three strategies of resource use: two species (*C. venosus* and *C. pellitus*) used the resource early in the season, both at the same period of the year and over a short interval of time. A third species (*C. elephas*) exploited the resource much later in the year, but also for a short time. Adults of the fourth species (*C. glandium*) were found on the trees throughout a more extended period. Once again, we did not find any significant difference in the timing of resource use between *C. venosus* and *C. pellitus*, whereas highly significant differences were found in all other pairwise comparisons (Figure 3, Table 1).

### Synchrony of the population dynamics

**Figure 4:**
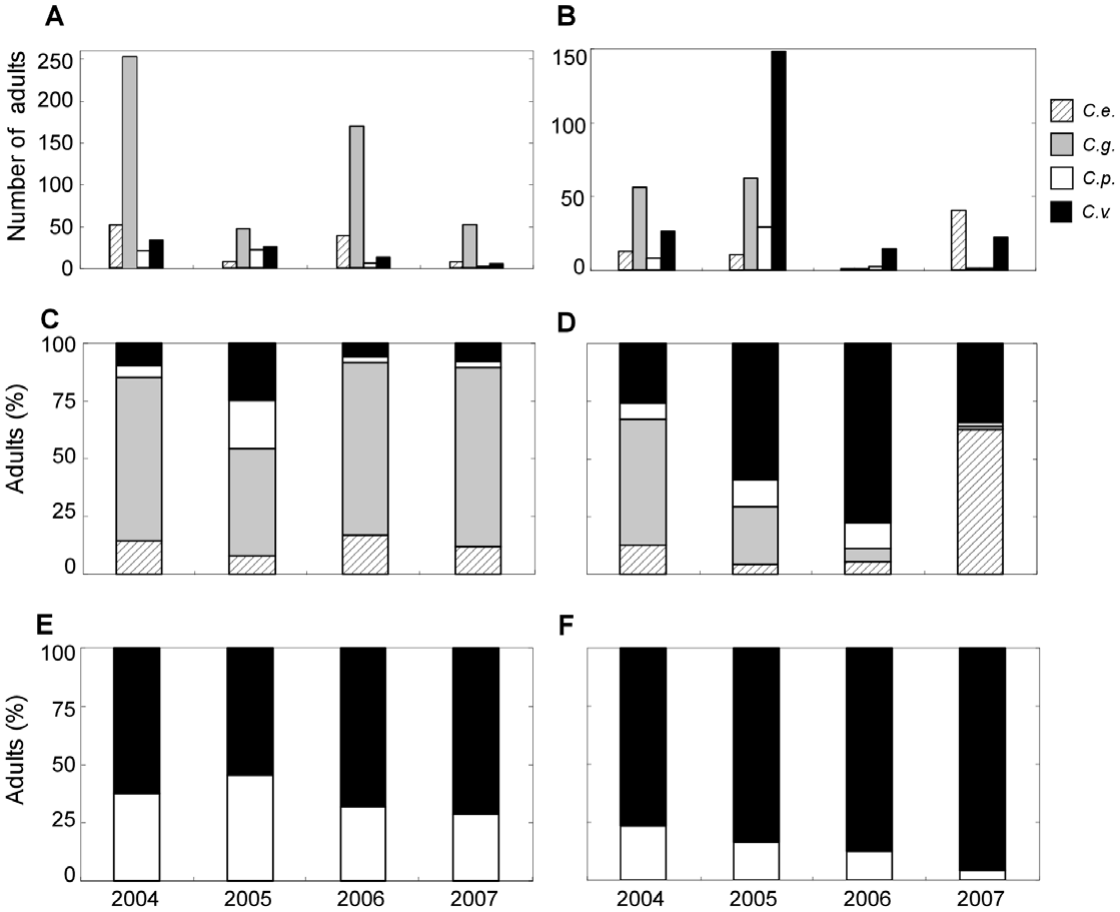
Asynchronous population dynamic of the four weevil species specialized on oak trees. We show the number of adult females of each species caught yearly from 2004 to 2007 on trees A and B (panels **A** and **B**, respectively) and their relative abundance on tree A (**C**) and tree B (**D**). *C. v*.: *Curculio venosus*; *C. p*.: *C. pellitus*; *C. g*.: *C. glandium*; *C. e*.: *C. elephas*. *C. venosus* and *C. pellitus* show no difference in their relative abundance (panels **E** and **F** for trees A and B, respectively), suggesting that these species are synchronized on both trees.

From these results, we expected the population dynamics of *C. venosus* and *C. pellitus* to be synchronized since they were the only ones that shared the same temporal pattern of resource use both within and between years. In contrast, we expected to find unsynchronized dynamics for all the other weevil species. In accordance with these expectations, we did indeed find that the four weevil species displayed overall unsynchronized population dynamics on both trees during the four-year survey (Figure 4; Tree A, Figure 4A,4C: 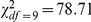, *p*<0.0001; Tree B, Figure 4B,4D: 
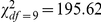
, *p*<0.0001). However, we did detect a strong, positive correlation between the population sizes of *C. venosus* and *C. pellitus* (see Figure 4E,4F, Table 1, R^2^ = 0.96), suggesting that these two species are synchronized. We did not find any significant correlation in any of the other pairwise comparisons between species (Table 1), which suggests that except for *C. venosus* and *C. pellitus*, the weevil species in the two communities surveyed exhibited unsynchronized population dynamics.

## Discussion

In the geographical area studied, four weevil sister species commonly co-occur on the same oak trees. Oak trees exhibit drastic between-year fluctuations in acorn production [29]–[30] (Figure 1). When the availability of a resource varies markedly across years, niche theory predicts that consumers' use of that resource will display clear time partitioning between species, and asynchronous dynamics [2], [17]–[18]. Conversely, the neutral theory predicts that the competing species, which are likely to be demographically equivalent, will display synchronized dynamics due to the strong “environmental forcing” [17]. From the survey of two independent communities belonging to different species living on two oak trees, we showed that the *Curculio* species generally did display marked time partitioning in their exploitation of the oak acorns, both within and between years, and that the same pattern was repeatedly observed in both the insect communities. These species also exhibited asynchronous population dynamics, suggesting that they responded in different ways to marked changes over time in the availability of the resource. These findings therefore match the predictions of the niche theory in a fluctuating environment [17]. However, in the two insect communities surveyed, *C. pellitus* and *C. venosus* never showed any difference in the timing of their resource use or in their population dynamics across years. Because their population dynamics are synchronized, probably by the pulses of availability of the resource, these two weevil species seem to be demographically equivalent [17]. Taken as a whole, therefore, our results do not fully match either the niche or the neutral theory of biodiversity. Instead, they provide empirical data consistent with recent theoretical developments suggesting a unifying theory of biodiversity [9], [11], [14]. In particular our empirical findings are among the first to support the theoretical proposal of Scheffer and van Nes [14], viz. that co-occurrence of species competing with each other is more likely between species that are either ecologically markedly distinct or very similar (see [39]–[40] for the two other recent empirical supports).

In agreement with niche theory, the asynchronous dynamics of species living in a fluctuating environment seem to result mainly from interspecific differences in the timing of their resource use. The remarkably constant time partitioning of the resource use between years exhibited by the competing species was due to their markedly divergent dormancy strategies (see Figure 2): adult emergence occurred either during the first or second years following larval development (*C. elephas*), during the second year only (*C. glandium*), or during the third year only (*C. venosus* and *C. pellitus*). Acorn crops produced by oak trees are characterized by a negative temporal autocorrelation since a mast year on a given tree is usually followed by a year in which that tree produces very little fruit. Mast years occur fairly regularly, *i.e*., every two to three years [29]–[30], which might favour *C. glandium* or *C. venosus* or *C. pellitus*. This regular periodicity is only partial, however, since the transannual pattern of the acorn crop also results from a random component. Of the four oak weevil species, *C. elephas*, is the only one in which adults from the same cohort commonly emerge over a period of more than one year: this species therefore seems to be the most efficient at coping with unpredictable fluctuations in resource availability by means of a bet-hedging strategy [35], [41], [42]. Consequently, because the occurrence of years of high resource abundance results from both predictable (negatively autocorrelated crop) and stochastic processes, the observed pattern of time partitioning between years in the weevil communities should favour different species from one pulse occurrence to the next, which should help to stabilize the coexistence of these four competing species.

In addition, the partitioning of the species within the year could also contribute to stabilize their coexistence since the amount of resources fluctuates from one year to the next. The level of competition for the resource varies from one year to the next, and seems to depend upon the number of oak acorns produced on one tree. Since there is a massive drop of unfertilized oak acorns in early summer [29], early-laying weevil species might suffer as a result of laying their eggs in acorns that may turn out to be unsuitable for larval development. These species would however be at an advantage as a result of their prior access to the resource when competition is high. Conversely, laying eggs late in the season might be the most efficient way of using the resource when competition is low, since only viable and mature fruits are available at that time, thereby ensuring safe larval development. The time partitioning within the year (see Figure 3) could favor early- and late-laying species during the years when competition is high and low, respectively, and thus could contribute to stabilizing the coexistence of competing weevil species. Whether the advantage of laying early differs from that of laying late, and whether the success of both strategies depends on the context of competition, yet remain to be investigated.

Since time partitioning of the resource can occur at two different timescales (within and between years), there may be many distinct temporal niches. Despite this, two of the four weevil species (*C. venosus* and *C. pellitus*) did not show any difference in their timing of resource use, either within or between years, and exhibited the same pattern at two independent sites. In addition, the fresh weight of the mature larvae emerging from the acorns did not differ between these two species either [43], which suggests that they are equally efficient at exploiting the resource. Although “absence of evidence is not evidence of absence”, these two weevil species do seem to be ecologically very similar. Because these two species are not phylogenetically closer to each other than to the other two weevil species described in our communities [31], we can safely exclude the phylogenetic constraint hypothesis to explain their ecological similarity. Because resource availability varied greatly year-to-year, we expected that these two “similar” species would exhibit similar demographic patterns. In accordance with this prediction, *C. venosus* and *C. pellitus* do indeed seem to respond the same way to the marked variations in resource availability over time. Two alternative conclusions can be drawn: first, their co-occurrence in the same communities could be driven by neutral dynamics only. Second, since these two weevil species are demographically nearly-equivalent, there could be minor differences in their ecological niches that would be sufficient to stabilize their coexistence. Such a “cryptic partitioning” could exist among drastically distinct categories of niches (e.g., the set of strategies developed to counter predation or parasites, or the use of different metabolic pathways to assimilate a resource), which could be too subtle to be detected in the system we were investigating. Nevertheless, should these two species co-occur neutrally or coexist in a stable way, we provide evidence that all the species belonging to the communities studied either harbor very distinct traits but with desynchronized dynamics, or are very similar both in their timing of the resource use and in their population dynamics.

Of course, a longer survey (*e.g.*, a 20-year survey at each site) would help to strengthen our conclusion. Meanwhile, examining data from long-term surveys of communities of other species competing for recurrent, pulsed resources would be the best way to reveal synchronized or desynchronized patterns of population dynamics. A 25-year survey is already available on annual plants competing for water in arid environments [16]. However, so far the results do not seem to have been considered in the light of the neutral theory of biodiversity. Interestingly, this study revealed that many, although not all, of the species investigated displayed both radically distinct ecological traits and unsynchronized population dynamics, thereby corroborating the niche theory. However, in line with our results, some plant species were found to be ecologically similar and to display synchronized population dynamics. From the study by Angert et al. [16], from our data, and despite a contrary previous interpretation, we propose that among species that are actually competing for the same resource in a strongly fluctuating environment, some might coexist despite being demographically very similar. Hence, the marked environmental fluctuations seem to favor the stable coexistence of “species groups” that are ecologically distinct from each other. Such environmental fluctuations would not preclude, however, the possibility that several ecologically-similar species could co-occur more neutrally within such a group [39], [44].

Although our system might be unusual owing to the marked variations of the environment with time, other species that are specialists for, and therefore compete for, recurrent pulsed resources appear to offer relevant models for community ecology studies, because they can be used to test theories of biodiversity in the field within a short time span. Despite pulsed resources are commonly encountered outside seeds produced by mast-seeding perennial plants or water in arid environments (see [21] for a review), the competitive coexistence of the communities of consumers of such resources has largely been ignored. Empirical results for such communities now need to accumulate which should stimulate further development toward a unified theory of biodiversity in fluctuating environments. To conclude, our work provides empirical evidence in favour of the counter-intuitive prediction that, in order to co-occur in a fluctuating environment, competing species must be ecologically either very diverse or very similar. Further theoretical development of both the neutral and the unified theories of biodiversity, which would notably relax the assumption of a constant environment and hence lead to more realistic predictions, is required before it is possible to propose a more general extrapolation from our findings.
